# The impact of self-monitoring physical and mental health via an mHealth application on postpartum weight retention: Data from the INTER-ACT RCT

**DOI:** 10.34172/hpp.42528

**Published:** 2024-03-14

**Authors:** Femke Geusens, Hanne Van Uytsel, Lieveke Ameye, Roland Devlieger, Yves Jacquemyn, Caroline Van Holsbeke, Annick Bogaerts

**Affiliations:** ^1^Department of Women’s and Children’s Health, Uppsala University, Uppsala, Sweden; ^2^REALIFE Research Group, Research Unit Woman and Child, Department of Development and Regeneration, KU Leuven, Leuven, Belgium; ^3^Department of Obstetrics and Gynecology, University Hospitals Leuven, Leuven, Belgium; ^4^Department of Obstetrics and Gynecology, Antwerp University Hospital UZA, Edegem, Belgium; ^5^Antwerp Surgical Training, Anatomy and Research Centre (ASTARC), Antwerp University, Antwerp, Belgium; ^6^Global Health Institute, Antwerp University, Antwerp, Belgium; ^7^Department of Obstetrics and Gynecology, Ziekenhuis Oost-Limburg ZOL, Genk, Belgium; ^8^Faculty of Health, University of Plymouth, Devon, UK

**Keywords:** Gestational weight gain, Obesity maternal, Telemedicine, Internet-based intervention, Psychosocial intervention

## Abstract

**Background:**

Postpartum weight retention (PPWR) has many health risks. Digital self-monitoring of weight can potentially make postpartum weight management easier. We aim to test to what extent the self-monitoring of weight, steps and mental health through an mHealth application increases postpartum weight loss and reduces the odds of substantial PPWR (≥5 kg).

**Methods:**

Participants were mothers in the intervention arm of the INTER-ACT multicenter randomized controlled trial (RCT), an inter-pregnancy lifestyle intervention among mothers with excessive gestational weight gain. Participants (n=288) had access to an mHealth application to log their weight, steps and mental health between 6 weeks and 6 months postpartum. A linear multiple regression model and a logistic regression model were run to test to what extent self-monitoring via the app increases postpartum weight loss and reduces the risk of substantial PPWR.

**Results:**

Women who logged their weight more often lost more weight (B=0.03, β=0.26, CI_B_ =[0.01,0.05], *P*<0.01), and had reduced odds of substantive PPWR (OR=0.99, CI_OR_ =[0.98, 0.999], *P*<.05). Mental health logging reduced the odds of substantive PPWR (OR=0.98, CI_OR_ =[0.97, 1.00], *P*<0.05), but was unrelated to the amount of weight loss. Steps logging was unrelated to either weight loss or substantive PPWR.

**Conclusion:**

Mothers with excessive gestational weight gain can benefit from app-based lifestyle interventions to reduce PPWR by self-monitoring their weight. More attention to mental health in PPWR interventions is needed.

## Introduction

 Postpartum weight retention (PPWR) refers to the inability to lose the weight gained during pregnancy after giving birth. There are several health risks associated with PPWR, such as an increased risk of long-term obesity,^1^ higher risk of chronic diseases such as cardiovascular diseases,^2^ an increased risk of complications in subsequent pregnancy,^3^ such as gestational diabetes mellitus,^4^ and mental health issues such as postpartum depression (PPD).^5^ This makes it important to support mothers with their postpartum weight management, as many women find it difficult to maintain a balance between the physical and emotional demands of the postpartum period and managing their weight.^1^ Lifestyle interventions can potentially support women in their postpartum weight management and reduce the risk of PPWR.

 Systematic reviews on PPWR and postpartum weight management show that lifestyle weight interventions can potentially reduce body weight in the postpartum period, but results are mixed and many questions regarding how to optimize interventions remain.^1,6^ Recently, apps, webpages or social media are being integrated in postpartum lifestyle interventions.^7^ These mHealth and eHealth interventions are still relatively new, and their effectiveness is still being assessed. One aspect that needs more scholarly attention, is the intensity with which mHealth applications are used to self-monitor behavior and feelings. It has been suggested that the effectiveness of mHealth interventions may be partially dependent on women’s self-monitoring.^7^

 Older studies from before the rise of mHealth interventions have demonstrated that self-monitoring is associated with weight loss in the general population.^8^ In these studies, self-monitoring was often done by hand using paper or electronic diaries and questionnaires. Digital self-monitoring using smartphone apps, with or without data from connected or ‘smart’ devices and wearables being automatically sent to a central app or monitoring page, can potentially make it easier for women to track their progress and reduce their risk of PPWR. Many commercial fitness and health apps already include such self-monitoring options through diet, steps or weight tracking,^9^ and pregnancy apps also include many self-monitoring tools, such as weight trackers, kick-counters, or bump trackers.^10^ However, our understanding of the extent to which app-based self-monitoring tools effectively improve health and wellbeing remains inconclusive.^11^

 We hypothesize that more intensive self-monitoring of weight and physical activity through the app increases the amount of weight lost between 6 weeks and 6 months postpartum (hypothesis 1) and reduces substantial PPWR at 6 months postpartum (hypothesis 2). In addition, the app also allows for the monitoring of mental health by logging emotional status and stress levels. Women with excessive gestational weight gain are vulnerable to mental health issues, and this in turn has been shown to increase PPWR and related body composition measures.^12^ However, there is little understanding of the role self-monitoring of mental health may have on reducing PPWR. Therefore, we question to what extent more intensive self-monitoring of emotional status and stress levels through the app increases postpartum weight loss (Research Question [RQ] 1) and reduces substantial PPWR (RQ2).

## Materials and Methods

###  Study design and participants

 Participants were drawn from the INTER-ACT (INTERpregnAncy Coaching for a healthy fuTure) multicenter RCT (for the protocol paper, see Bogaerts et al).^13^ The INTER-ACT intervention focuses on mothers with excessive gestational weight gain. Excessive gestational weight gain is an important risk factor for PPWR as well as several other adverse pregnancy and neonatal outcomes.^14^ The main aim of the intervention is to reduce pregnancy-induced hypertension, gestational diabetes mellitus, need for caesarean-section and large-for-gestational-age babies in the following pregnancy.^13^ To reduce these risks, the intervention aims to encourage healthy lifestyle changes in the mothers, with tailored attention to personal barriers and how to overcome these barriers.^13^ The intervention consists of two phases: the first intervention phase (the postpartum phase) runs between 6 weeks and 6 months postpartum, and the second intervention (the new pregnancy phase) phase runs during the next pregnancy.^13^ In these phases, mothers receive face-to-face coaching supported by a mobile application which they can use for self-monitoring of weight, physical activity, emotional status and stress level.

**Figure 1:**
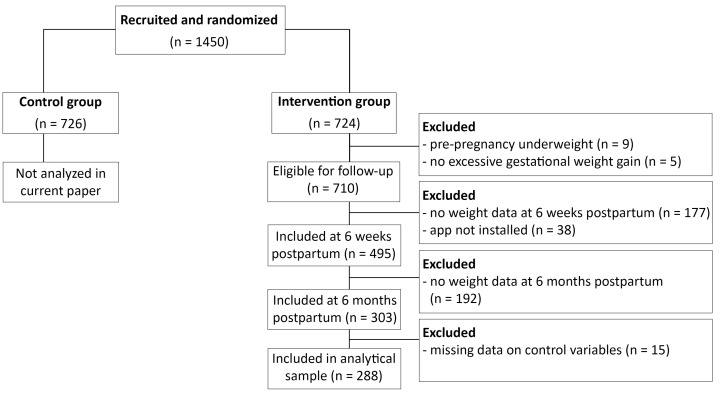
Flowchart of the recruitment and inclusion of participants

 Data were collected between May 2017 and July 2019. Participants were recruited a few days after giving birth by study midwives in 6 hospitals in Flanders. Women were eligible for participation if they were at least 18 years old, had excessive gestational weight gain in line with the gestational weight gain guidelines of the Institute of Medicine guidelines,^15^ did not require complex medical diets, did not have a history of bariatric surgery or planned on getting one in the near future, had no chronic disorders or significant psychiatric disorders, and had not had a stillbirth in the last pregnancy. See Figure 1 for a flowchart of the recruitment and inclusion of participants. All participants in the intervention group who met the inclusion criteria and had no missing data were included, resulting in an analytical sample of n = 288. Please see the protocol paper for the full sample size calculation.^13^ In the current study, we conducted secondary analysis on data from the intervention group from the first phase of the trial: from 6 weeks postpartum until 6 months postpartum. The control group was not included in this sub-study, because they did not have access to the app.

###  The intervention and smartphone app

 Participants were randomized into the intervention group or control group within a week after recruitment. Individuals in the intervention group received four face-to-face lifestyle coaching sessions at approximately 6 weeks, 8 weeks, 12 weeks and 6 months postpartum. In these sessions, women worked on topics related to the impact of lifestyle on health and wellbeing. Women were coached on food intake and nutrition, physical activity, stressors and social support. They received tailored informational education and decision-making related coaching with regard to these topics, through shared-decision making frameworks, motivational interviewing and goal setting. To standardize the coaching sessions, all coaches received a detailed manual including the steps, techniques and content to discuss in each session.

 In addition, a smartphone application was designed for this study and installed on the participants’ phone during the first appointment. The aim of this smartphone app was to support the participants in their behavior change between the face-to-face coaching sessions. In the app, they could track their weight using a Bluetooth-connected weighing scale (Withings Body + ), their steps using a Bluetooth-connected pedometer (Withings Go), and their mental health by manually entering their current emotional status and stress level. Women could set goals in the app and track their goals by tracking their weight, physical activity and mental health. The coaches could see the women’s app input, and send personalized motivational messages and informational tips to reach their goals based on the women’s input. App use was also discussed in the coaching sessions. More details on the intervention and app design can be found in the study protocol. ^13^

###  Measurements

####  Intensity of app use

 We counted how often a participant logged her weight, steps, emotional status and stress level between 6 weeks postpartum and 6 months postpartum. Due to multicollinearity issues (*r* = 0.98, *P* < 0.001), emotional status and stress level logs had to be combined in one ‘mental health’ log measure, using a mean score. Overall, a higher score reflects more intensive app use.

###  Weight loss at 6 months postpartum

 Because participants could start using the app at 6 weeks postpartum, and we were interested in the unique effect of using the app, weight loss at 6 months postpartum was calculated by subtracting the participant’s weight at 6 months postpartum from their weight at 6 weeks postpartum.

###  Substantial postpartum weight retention at 6 months postpartum

 Substantial PPWR was measured by subtracting the pre-pregnancy weight from the weight at 6 months postpartum. Participants who had retained at least 5 kg were classified as substantial PPWR.^16,17^

###  Control variables

 With regard to weight covariates, we measured gestational weight gain by subtracting the self-reported pre-pregnancy weight from the weight at birth, which was measured in the hospital at time of birth. Then, the maximum recommended weight gained was subtracted from the gestational weight gain measure to obtain the amount of excessive gestational weight gain. Weight loss at 6 weeks postpartum - at the start of the intervention - was calculated by subtracting the weight at 6 weeks postpartum (weighed by the study nurse at intake) from the weight at birth. Pre-pregnancy body mass index (BMI) was calculated using self-reported pre-pregnancy weight/length^2^. History of depression was measured by asking participants whether they had suffered from depressive symptoms in the past (yes = 1, no = 0), and history of anxiety was measured by asking participants whether they had suffered from intense feelings of anxiety in the past (yes = 1, no = 0). We included participants’ age at the birth of their child. Multiparity was calculated by asking mothers how many children they had, included the baby they had just given birth to, which was recoded into primiparous ( = 0) or multiparous ( = 1), participants were asked how they were feeding their child at 6 weeks postpartum, whereby exclusive breastfeeding and a combination of breastfeeding and bottle-feeding was recoded into breastfeeding ( = 1) and exclusive bottle-feeding with or without prior breastfeeding was recoded into not breastfeeding at 6 weeks postpartum ( = 0). Finally, the time between the 6-week and 6-month measurements was calculated by subtracting the 6-week appointment date from the 6-month appointment date and dividing this by 7 to calculate the weeks between measurements.

###  Statistical analyses

 SPSS version 28^18^ was used to calculate the descriptive statistics. To test hypothesis 1 and RQ1, we used Mplus version 8^19^ to test a multiple regression model in which we regressed weight lost between 6 weeks and 6 months postpartum on the amount of weight logs, steps logs and mental health logs between intake (6 weeks postpartum) and follow-up. We controlled for the amount of excessive gestational weight gained, pre-pregnancy BMI, postpartum weight lost before intake, history of depression, history of anxiety, age of the mother at birth, multiparity, breastfeeding at 6 weeks postpartum and the number of weeks between intake and follow-up. Correlations were modelled between all independent variables, with the exception of weeks between measurements^[1]^. The model was estimated using a maximum likelihood parameter with robust standard errors (MLM) to account for potential non-normality of the data. Model fit was decided upon inspection of the comparative fit index (CFI, > 0.95), Tucker-Lewis index (TLI, > 0.95), root mean square error of approximation (RMSEA, < 0.06), and standardized root mean squared residual (SRMR, < 0.08).^20^ Finally, to test hypothesis 2 and RQ2, a logistic regression was run in Mplus in which we predicted the odds of having a substantial PPWR ( ≥ 5 kg) based on the aforementioned predictor and control variables in the multiple regression. All variables were entered as manifest variables.

## Results

###  Participants’ characteristics

Table 1 shows an overview of the participants’ characteristics. First, with regard to app use, 6.9% (n = 20) of our participants did not use the app at all, 11.5% did not log their weight, 12.8% did not log their steps, and 11.1% did not log their mental health. On average, participants logged their weight 32 times between 6 weeks and 6 months postpartum, their steps 56 times and their mental health 18 times. Only one person logged their mental health more than 100 times, 6.6 % (n = 19) logged their weight at least 100 times and 19.4% (n = 56) logged their steps at least 100 times. Participants who logged their weight, steps or mental health at least 100 times were considered heavy users.

**Table 1:** Participant characteristics

**Variable **	**Full analytical sample**		**Stratified by app use**	
	**(n=288)**	**No use (n=20)**	**Light use** ^a^ ** (n=205)**	**Heavy use** ^a^ ** (n=63)**
	**Mean (SD) [%]**	**Mean (SD) [%]**	**Mean (SD) [%]**	**Mean (SD) [%]**
Intensity of app use between 6 weeks & 6 months postpartum				
Weight logs [% none]	31.91 (33.57) [11.5]	0 [100.0]	25.32 (22.46) [6.3]	63.49 (45.50) [0.0]
Steps logs [% none]	56.15 (44.94) [12.8]	0 [100.0]	42.80 (28.94) [8.3]	117.44 34.15) [0.0]
Mental health [% none]	18.51 (21.67) [11.1]	0 [100.0]	14.98 (16.17) [5.4]	35.86 (29.27) [1.6]
Weight fluctuations				
Total gestational weight gain	17.77 (4.15)	17.25 (4.80)	17.96 (4.28)	17.36 (3.45)
Excessive gestational weight gain	4.51 (4.05)	3.88 (4.99)	4.74 (4.00)	3.97 (3.88)
Postpartum weight loss at 6 weeks postpartum [% weight gained]	10.74 (3.41) [0.3]	9.70 (2.88) [0.0]	10.84 (3.63) [0.5]	10.73 (2.75) [0.0]
Postpartum weight loss between 6 weeks and 6 months postpartum [% weight gained]	2.42 (4.12) [23.6]	1.74 (4.14) [20.0]	2.13 (3.78) [23.9]	3.57 (4.95) [23.8]
PPWR at 6 months postpartum [% substantial] ^b^	4.62 (5.12) [44.4]	5.77 (5.44) [55.0]	4.99 (4.97) [46.8]	3.06 (5.15) [33.3]
Pre-pregnancy BMI [% normal]	25.61 (4.23) [47.9]	25.90 (5.04) [50.0]	25.61 (4.24) [46.8]	25.54 (4.01) [50.8]
Control variables				
Age [% 35 or older]	30.53 (3.89) [12.5]	31.15 (4.17) [20.0]	30.44 (3.83) [17.1]	30.65 (4.07) [17.5]
Multiparous, n [%]	129 [44.8]	13 [65.0]	89 [43.4]	27 [42.9]
Breastfeeding at 6 weeks postpartum, n [%]	199 [69.1]	15 [75.0]	136 [66.3]	48 [76.2]
History of depression, n [%]	37 [12.8]	4 [20.0]	33 [16.1]	0 [0.0]
History of anxiety, n [%]	31 [10.8]	4 [20.0]	25 [12.2]	2 [3.2]

Note: ^a^ light use is defined as logging weight, steps or mental health between 1 and 99 times, and heavy use is defined as logging weight, steps or mental health at least 100 times, ^b^ substantial PPWR is defined as PPWR ≥ 5 kg.

 Women weighed on average 71.6 kg before pregnancy (SD = 12.42; BMI = 25.61, SD = 4.23) and 89.4 kg at birth (SD = 11.78). They had an average gestational weight gain of 17.8 kg (SD = 4.15), which correspond to an average excessive gestational weight gain of 4.5 kg (SD = 4.05). At 6 weeks postpartum, they weighed on average 78.7 kg (SD = 12.50) and had lost on average 10.7 kg (SD = 3.41) after childbirth. At 6 months postpartum, they weighed on average 76.2 kg (SD = 13.53) and lost an additional 2.4 kg (SD = 4.11) compared to 6 weeks postpartum. One woman gained weight between childbirth and 6 weeks postpartum and nearly one in four women (23.6%) gained weight between 6 weeks postpartum and 6 months postpartum. At 6 months postpartum, women had an average PPWR of 4.6 kg (SD = 5.12); 17.4% had no PPWR and were back at their pre-pregnancy weight or lower, and 44.4% had PPWR of at least 5 kg.

###  The role of self-monitoring in postpartum weight loss and PPWR

 The main model had excellent fit (χ^2^ (df) = 11.84 (11), *P* = 0.38, CFI = 0.98, TLI = 0.98, RMSEA = 0.02, SRMR = 0.02). See Table 2 for the full results of the multiple regression model including the control variables. Weight loss at 6 months postpartum compared to 6 weeks postpartum was significantly and positively predicted by the frequency of logging weight in this period (B = 0.03, *P* = 0.007). In contrast, logging steps or logging mental health did not significantly predict weight loss. Thus, hypothesis 1 is partially confirmed: self-monitoring weight, but not steps, increases weight loss. To answer RQ1: more intensive self-monitoring of mental health is unrelated to postpartum weight loss.

**Table 2:** Results of the multiple regression of intensity of app use predicting weight loss at 6 months postpartum

**Predictor**	Weight loss between 6 weeks and 6 months postpartum, R^2^=0.22***			
	**B(SE)**	**β**	**Z**	**95% CI**
Intercept	10.22 (3.33)**	2.49	3.07	[-1.67, 11.64]
Weight logs between 6 weeks & 6 months postpartum	0.03 (0.01 )**	0.26	2.69	[0.01, 0.05]
Steps logs between 6 weeks & 6 months postpartum	-0.01 (0.01)	-0.06	-0.91	[-0.02, 0.01]
Mental health logs between 6 weeks & 6 months postpartum	0.01 (0.01)	0.07	1.17	[-0.01, 0.04]
Pre-pregnancy BMI	-0.35 (0.06)***	-0.36	-5.53	[-0.47, -0.22]
Excessive gestational weight gain	0.21 (0.06)***	0.21	3.75	[0.10, 0.33]
Weight lost at 6 weeks postpartum	-0.27 (0.11)*	-0.23	-2.48	[-0.49, -0.06]
History of depression^a^	-0.83 (0.63)	-0.07	-1.32	[-2.08, 0.41]
History of anxiety^a^	-0.01 (0.63)	-0.001	-0.02	[-1.25, 1.23]
Age	-0.01 (0.07)	-0.01	-0.20	[-0.14, 0.12]
Multiparous^a^	1.10 (0.49)*	0.13	2.25	[0.14, 2.06]
Breastfeeding at 6 weeks postpartum^a^	-0.30 (0.48)	-0.03	-0.63	[-1.23, 0.63]
Weeks between week 6 and month 6 weight measurements	0.12 (0.07)	0.08	1.73	[-0.02, 0.25]

Note: * *P* < 0.05, ** *P* < 0.01, *** *P* < 0.001, ^a^ dichotomous variable (0 = no, 1 = yes). Statistically significant values were shown in bold for quick interpretation.

 An additional logistic regression model (Table 3) confirmed that participants had significantly lower odds of substantial PPWR if they used the app more intensively for self-monitoring weight (OR = 0.98, *P* = 0.036). Self-monitoring mental health was also associated with reduced odds of substantial PPWR (OR = 0.98, *P* = 0.045), but self-monitoring steps was not. Thus, hypothesis 2 is partially confirmed: more intensive self-monitoring of weight, but not of steps predicts reduced odds of substantial PPWR. With regard to RQ2, we can conclude that more intensive self-monitoring of mental health was associated with reduced odds of substantial PPWR.

**Table 3:** Results of the logistic regression of intensity of app use predicting the odds of high postpartum weight-retention at 6 months postpartum

**Predicto**r	**Substantial PPWR at 6 months postpartum**			
	**B (SE)**	**β**	**OR**	**95% CI OR**
Weight logs between 6 weeks & 6 months postpartum	-0.01 (0.01 )*	-0.14	0.99	[0.98, 0.999]
Steps logs between 6 weeks & 6 months postpartum	0.01 (0.004)	0.10	1.01	[.998, 1.02]
Mental health logs between 6 weeks & 6 months postpartum	-0.02 (0.01)*	-0.14	0.98	[0.97, 1.00]
Pre-pregnancy BMI	-0.08 (0.04)	-0.13	0.92	[0.85, 1.001]
Excessive gestational weight gain	0.40 (0.06)***	0.58	1.50	[1.33, 1.69]
Weight lost at 6 weeks postpartum	-0.50 (0.07)***	-0.60	0.61	[0.53, 0.70]
History of depression^a^	1.18 (0.52)*	0.14	3.27	[1.17, 9.09]
History of anxiety^a^	0.52 (0.56)	0.06	1.68	[0.56, 5.01]
Age	0.003 (0.04)	0.004	1.00	[0.92, 1.09]
Multiparous^a^	-0.5 (0.34)	-0.09	0.61	[0.31, 1.18]
Breastfeeding at 6 weeks postpartum^a^	0.22 (0.36)	0.04	1.24	[0.62, 2.50]
Weeks between measurements	-0.09 (0.06)	-0.09	0.91	[0.81, 1.03]

Note: * *P* < 0.05, ** *P* < 0.01, *** *P* < 0.001, ^a^ dichotomous variable (0 = no, 1 = yes). Statistically significant values were shown in bold for quick interpretation.

###  Significant correlations between the independent variables in the model

 See Table 4 for an overview of all model-estimated correlations between the independent variables as modelled in the multivariate multiple regression. Weight logging between 6 weeks and 6 months postpartum correlated significantly and positively with mental health logging and steps logging, as well as negatively with history of depression and history of anxiety. Steps logging correlated significantly and positively with mental health logging, and negatively with history of depression and history of anxiety. Mental health logging correlated significantly and negatively with history of depression and positively with age. Finally, weight loss at 6 weeks postpartum correlated positively with excessive gestational weight gain, and negatively with pre-pregnancy BMI and being multipara; pre-pregnancy BMI correlated positively with history of depression and excessive gestational weight gain; and finally, age correlated positively with breastfeeding and being multiparous. No other associations were significant.

**Table 4:** Standardized model-estimated correlations between the independent variables in the multiple regression model

**Variable**	**1.**	**2.**	**3.**	**4.**	**5.**	**6.**	**7.**	**8.**	**9.**	**10.**
1. Weight logs										
2. Steps logs	0.47***									
3. Mental health logs	0.33***	0.52***								
4. Pre-pregnancy BMI	-0.06	-0.06	-0.09							
5. Excessive gestational weight gain	-0.03	-0.07	-0.08	0.27***						
6. Weight lost at 6 weeks postpartum	0.04	0.07	0.08	-0.25***	0.23***					
7. History of depression	-0.11*	-0.18***	-0.13***	0.15*	0.12	-0.08				
8. History of anxiety	-0.14***	-0.16***	-0.05	0.08	0.04	-0.11	0.34***			
9. Age	0.03	0.02	0.11*	0.07	-0.09	-0.05	0.10	0.08		
10. Multiparous	-0.02	0.02	0.03	0.04	-0.10	-0.18**	0.05	-0.07	0.28***	
11. Breastfeeding at 6 weeks postpartum	0.09	0.04	-0.03	-0.04	-0.10	0.02	-0.10	-0.01	0.19***	0.07

*Note:* * P < 0.05, ** *P* < 0.01, *** *P* < 0.001.

## Discussion

 In this study, we aimed to test to what extent the intensity of self-monitoring of physical and mental health using an mHealth application can affect postpartum weight trajectories. The women in our study all had excessive gestational weight gain. As a result, many of them struggled with PPWR. On average, they retained 4.6 kg, which is just barely under the 5 kg cut-off for substantive PPWR.^21,22^ Moreover, almost half our sample had substantive PPWR and one in four gained weight between 6 weeks and 6 months postpartum. Considering that all of the women in our sample were part of an intervention to reduce PPWR, this shows that even with help, women with excessive gestational weight gain clearly struggle to reduce their PPWR. This reinforces the importance of designing interventions that target women with excessive gestational weight gain, as prior research has also shown that gestational weight gain is an important risk factor of PPWR.^21,23^

 Lifestyle interventions have the potential to reduce PPWR, but the evidence for their effectiveness is mixed.^1,6^ It is thus important for research to elucidate which components work best. We found that adding mHealth self-monitoring functions can increase the effectiveness of postpartum lifestyle interventions. Overall, the INTER-ACT postpartum intervention has previously been shown to have limited effectiveness: It improved postpartum eating behavior, but not physical behavior,^24^ nor did it have a direct effect on PPWR, fat percentage or waist circumference changes.^25^ However, women who changed their eating behavior as a result of the intervention did retain less postpartum weight, and decreased more of their fat percentage and waist circumference than women who had not changed their eating behavior.^25^ This shows the importance of not only directly measuring outcomes (e.g., weight or body composition), but also the behavioral change mechanisms predicting these outcomes (e.g., eating behavior). We now add to this knowledge that the implementation of app-based self-monitoring may be one such behavioral change mechanisms that is useful to increase postpartum weight loss and decrease substantial PPWR.

 Self-monitoring has previously been demonstrated to be an effective behavioral change technique, but this has often been studied in a low-digital context with manual logging.^8,26^ The proliferation of connected or so-called ‘smart’ devices can potentially make self-monitoring easier, thus increasing adherence to the intervention. In particular, the postpartum period is a challenging period in a new parent’s life, and many struggle with finding the time to take care of themselves.^1,27^ Postpartum women indicate to be open to mHealth and eHealth lifestyle interventions, and believe them to be convenient and practical.^27^ They want to be able to monitor their mental and physical health,^27,28^ and automating these tracking features through connected devices can potentially increase usability by lowering the effort that is needed to self-monitor. Indeed, we saw that the most automated function in our app (steps tracking via the connected pedometer) was the most frequently used tracking function, whereas the least automated function (mental health tracking through answering mini-surveys) was the least used function.

 However, although the automation of the tracking can increase the ease of use, it does not necessarily increase the effectiveness. In particular, we found that the easiest-to-use tracker which was also the most frequently used (the steps tracker) was the least effective. It is likely that this tracker was more of a background function. Steps were automatically logged by the pedometer and sent to the app, without much effort or intervention of the participants. So while it did log a woman’s steps, she may not be using this function to actively self-monitor her level of physical activity. In contrast, we found that using the app to monitor weight did facilitate postpartum weight loss and reduces substantial PPWR. Although the logging of the weight in the app is automated, the woman needs to step on the weighing scale in order for her weight to be measured and logged. This implies a more active choice to self-monitor.

 Our finding that the more often women weighed themselves, the more weight they lost over the intervention period and the less likely they were to have substantial PPWR at 6 months postpartum is in line with prior research on paper-based self-monitoring in the general population^8^ and a slightly older meta-analysis on effective strategies for weight loss in the postpartum period before the rise of mHealth app-based interventions.^26^ However, it should be noted that it is also possible that women who found it easier to lose weight during the intervention period where more motivated to continue weighing themselves and monitoring their weight. It is possible that as women noticed that weight loss was harder than expected, they were less likely to continue monitoring their weight. Nevertheless, women who had already lost more weight before the start of the intervention, or who had gained less excessive gestational weight during their pregnancy were not using the weight logging function more frequently than women with less postpartum weight loss before the start of the intervention or higher excessive gestational weight gain. This leads us to believe that it is probable that weight logging via a smartphone application can indeed assist women in their weight loss journey. Active self-monitoring of weight should be encouraged, and smartphone apps with connected devices can potentially make this self-monitoring easier and more user friendly.

 Our results further demonstrate the importance of mental health in PPWR and lifestyle interventions. Women with prior depression were three times more likely to have substantial PPWR compared to women without prior mental health issues. Prior research has found mixed results on the role of mental health and depression on PPWR,^29-31^ but our results point in the direction of mental health being an important factor. Thus, it seems important to integrate mental health components in PPWR interventions. In this study, we found that the self-monitoring of mental health did reduce the risk of substantial PPWR but did not significantly increase the amount of weight loss during the intervention period. Consequently, while our results point towards the integration of mental health components in PPWR interventions, women may need more than just self-monitoring. More therapy-based interventions that work on the root causes of their mental health issues may potentially be valuable,^32^ and the self-monitoring of mental health can here be used as a tool to assist patient-provider conversations.

 Furthermore, mental health struggles seem to not only form a hurdle to reduce PPWR, but also to intervention adherence. Women with a history of mental health issues use the app self-monitoring functions less intensely. For these women, the app seems less attractive than for women without a history of mental health issues. It is unclear why this may be, but there are some potential explanations that may be interesting to explore in future research. For example, it is possible that women with a history of mental health issues are less intrinsically motivated to self-monitor their mental and physical health. It is possible that self-monitoring is confrontational for these women, or cause prior feelings of anxiety to flare up again.^33^ It is also possible that these women have previously worked with mental health professionals or tried self-help techniques, and they have reached a point where they are fed up with the idea of ‘improving themselves’ because they have done so excessively in the past already, thus reducing their motivation to self-monitor.^34^ These potential explanations should be further explored in qualitative research, but our results do suggest that mHealth interventions may be less interesting to reach women with prior mental health struggles. Considering the link between mental health struggles and PPWR^12^ it would be beneficial to co-design future interventions together with this target group to ensure that the intervention meets their needs.

 Finally, our findings indicate a need for a more holistic approach to PPWR that starts much earlier than the postpartum period. The abovementioned history of depression is an important predictor of substantive PPWR, and so are pre-pregnancy BMI and the amount of excessive weight gained during the pregnancy. This indicates a need to prepare women for a healthy postpartum period even before they get pregnant. Prevention is generally easier than cure,^35^ especially considering the extra barriers for healthy living that arise during the postpartum period.^1^

## Limitations and suggestions for future research

 There was extensive attrition (65.1%) between enrollment in the study and completion of the 6 month-follow up measurement. Some of the main motivations for dropping out that were mentioned, were a lack of time, finding it easy to return to the pre-pregnancy weight without additional help, and finding it difficult and frustrating to lose weight. A more in-depth study into the motivations for dropping out may provide more insight in how to develop our interventions to be more in line with women’s expectations. In addition, more research is needed on how to motivate women to self-monitor their weight in the postpartum period.

 In addition, the mHealth application was integrated in a broader intervention which also included face-to-face coaching sessions. Because only the women in the intervention arm of this intervention received access to the app, we could not compare the effectiveness of the app between women who also received coaching and women who did not receive coaching. It is possible that the coaches encouraged mothers to use the app more frequently than they would have done on their own without a coach. Future research would benefit from a purified design focusing on digital tools, in which app-based self-monitoring is the focus and compared to a control group, instead of only part of the intervention design.

 Finally, the app was designed specifically for this study, but there were some technical issues throughout the intervention. There were moments where the app was unavailable to the women or crashed repeatedly, which may have discouraged women from continuing to use the app. It would be interesting to repeat this study using commercially available apps in a more natural setting outside of an official intervention setting, to test the effectiveness of app-based self-monitoring for postpartum weight management.

## Conclusion

 Mothers with excessive gestational weight gain can benefit from app-based lifestyle interventions to reduce PPWR in which they are encouraged to self-monitor their weight. Self-monitoring of other health aspects, such as steps and mental health, seems less relevant in such interventions, though more attention is needed for the role of mental health in postpartum weight management interventions as a whole. Based on our results, we encourage healthcare providers to implement app-based self-monitoring of weight in their postpartum care of women with excessive gestational weight gain.

## Acknowledgements

 We would like to thank the study midwives who helped with the recruitment for this study, as well as FWO Vlaanderen, Rotary Foundation Limburg, Horizon Europe, Agentschap Kind en Gezin/Opgroeien (Flemish governmental agency in charge of preventive health care for young children), and Wit-Gele Kruis Limburg (Flemish organization for at-home nursing and midwifery care) for their financial and practical support.

## Competing Interests

 The authors have no conflict of interest to report.

## Endnotes

 [1] There were no significant zero-order correlations between days between measurements and the other independent variables, and modeling correlations between this variable and the other independent variables resulted in an overfitted model.

## Ethical Approval

 This study was approved by the Clinical Trial Center/Ethical Committee UZ Leuven (protocol code B322201730956/S59889).
